# Impact of High Immunoreactivity on Surgically Treated Serum p53 Antibody-Positive Esophageal Squamous Cell Carcinoma after Neoadjuvant Therapy: A Multi-Institutional Study of 637 Cases

**DOI:** 10.5761/atcs.oa.26-00142

**Published:** 2026-09-10

**Authors:** Takashi Suzuki, Satoshi Yajima, Akihiko Okamura, Yusuke Taniyama, Yu Ohkura, Koichi Yagi, Takashi Fukuda, Ryo Ogawa, Yasuhiro Tsubosa, Hideaki Shimada

**Affiliations:** 1 Department of Gastroenterological Surgery and Clinical Oncology, Graduate School of Medicine, Toho University, Tokyo, Japan; 2 Department of Gastroenterological Surgery, Gastroenterology Center, The Cancer Institute Hospital of Japanese Foundation for Cancer Research, Tokyo, Japan; 3 Department of Digestive Surgery, Tohoku University Graduate School of Medicine, Sendai, Miyagi, Japan; 4 Department of Gastroenterological Surgery, Toranomon Hospital, and Okinaka Memorial Institute for Medical Research, Tokyo, Japan; 5 Department of Gastrointestinal Surgery, Graduate School of Medicine, The University of Tokyo, Tokyo, Japan; 6 Department of Gastroenterological Surgery, Saitama Cancer Center Hospital, Kitaadachi-gun, Saitama, Japan; 7 Department of Gastroenterological Surgery, Nagoya City University Graduate School of Medical Sciences, Nagoya, Aichi, Japan; 8 Division of Esophageal Surgery, Shizuoka Cancer Center, Suito-gun, Shizuoka, Japan

**Keywords:** esophageal squamous cell carcinoma, serum p53 antibody titers, neoadjuvant chemotherapy

## Abstract

**Purpose:**

This multi-institutional study investigated the clinical and prognostic significance of serum p53 antibody (s-p53-Ab) titers in patients with s-p53-Ab-positive esophageal squamous cell carcinoma (ESCC) treated with neoadjuvant chemotherapy (NAC).

**Methods:**

We analyzed 637 patients who underwent surgery at 15 institutions. Patients were divided into upfront surgery (n = 375) and NAC (n = 262) groups and stratified into high-titer (≥9.82 ng/mL) and low-titer (>1.30 to <9.82 ng/mL) groups.

**Results:**

In the upfront surgery group, high-titer patients had significantly worse relapse-free survival (P = 0.046) and overall survival (P = 0.024) than low-titer patients. In contrast, no significant survival differences were observed by titer after NAC, and the poorer prognosis associated with high s-p53-Ab titers in the upfront surgery cohort was not seen after NAC. Subgroup analyses of stage II and III disease showed similar, nonsignificant trends toward improved outcomes in high-titer patients after NAC.

**Conclusions:**

These findings suggest that high s-p53-Ab titers are associated with a poor prognosis in patients undergoing upfront surgery. Although these observations did not reach statistical significance, patients with high titers may potentially benefit from NAC, indicating its potential therapeutic value in those with a strong p53 antibody response.

## Abbreviations

ESCC
: esophageal squamous cell carcinoma
s-p53-Abs
: serum p53 antibodies
CF
: cisplatin and 5-fluorouracil
NAC
: neoadjuvant chemotherapy

## Introduction

Esophageal squamous cell carcinoma (ESCC) is highly prevalent in East Asia, South-Central Asia, and South Africa, accounting for approximately 85% of all esophageal cancer cases.^1)^ In Japan, squamous cell carcinoma constitutes approximately 90% of all esophageal cancers.^2)^ To improve treatment outcomes, various multidisciplinary clinical trials that involve surgery, chemotherapy, and radiotherapy have been conducted. The JCOG9204 trial revealed the efficacy of adjuvant chemotherapy with cisplatin and 5-fluorouracil (CF) compared with surgery alone.^3)^ Subsequently, the JCOG9907 trial demonstrated the superiority of neoadjuvant chemotherapy (NAC) over adjuvant chemotherapy.^4)^

Serum p53 antibodies (s-p53-Abs) are tumor markers that reflect p53 gene mutations within cancer cells. We have previously revealed the clinicopathological significance of s-p53-Abs in patients with ESCC.^5–8)^ A nationwide multi-institutional study by the Japan Esophageal Society showed that, among patients who underwent upfront surgery, those with high s-p53-Ab titers achieved significantly poorer prognoses compared with those having low titers, even in s-p53-Ab-negative cases.^7)^

However, to date, no studies have exclusively focused on s-p53-Ab-positive cases or on antibody titers, which may reflect the host immune response. Moreover, no reported multi-institutional studies have specifically assessed s-p53-Abs in patients receiving NAC—the current standard of care for advanced ESCC.^8,9)^ This multi-institutional collaborative study aimed to investigate the prognostic significance of s-p53-Ab titers in patients with s-p53-Ab-positive ESCC treated with NAC. Previous reports have described the diagnostic use of s-p53-Abs in ESCC; however, their association with chemotherapy response remains unclear.

## Patients and Methods

This retrospective multicenter study included 3003 consecutive patients with primary ESCC who underwent curative esophagectomy with regional lymph node dissection at 15 Japanese institutions between 2008 and 2016. Among the 2361 cases available for evaluation, 1487 patients underwent curative surgery without preoperative treatment (upfront surgery), and 874 patients received NAC with fluorouracil and cisplatin. Among the 637 s-p53-Ab-positive cases, 375 underwent upfront surgery (25.2%) and 262 received NAC (30.0%). The NAC group showed a significantly higher s-p53-Ab positivity rate (p = 0.013).

The cohort comprised 1987 men (84.2%) and 374 women (15.8%), with a median age of 66 years (range, 30–92 years). Pre-treatment clinical staging was performed following the eighth edition of the Union for International Cancer Control (UICC) TNM classification system for esophageal cancer^10)^ and included patients with stages I (n = 868), II (n = 604), III (n = 724), and IV (n = 165) diseases.

**Fig. 1:**
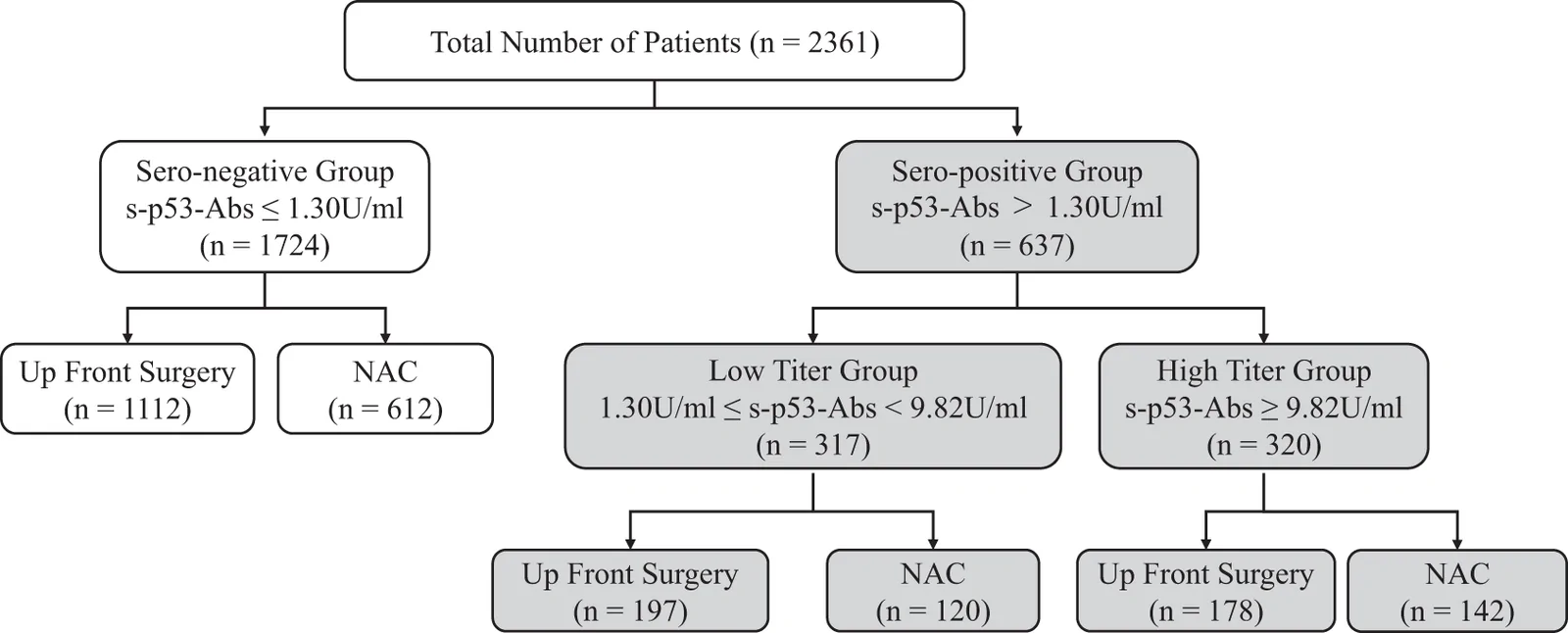
Flowchart illustrating the patient selection for the study.

Among the overall cohort, 637 patients tested positive for s-p53-Abs, comprising 375 patients who underwent upfront surgery and 262 who received NAC (**Fig. 1**). Of the patients positive for s-p53-Ab, 537 (84.3%) were men and 100 (15.7%) were women, with a median age of 66 years (range, 36–92 years). Clinical staging distribution in this subgroup was stages I (n = 198), II (n = 173), III (n = 212), and IV (n = 54).

The Ethics Committee of the Faculty of Medicine, Toho University (Approval No. A18112_A17044_A16037) and the participating institution approved this study. Information about the study was posted on the institution’s website, and potential participants were free to opt out.

### Follow-up and recurrence evaluation

The median follow-up duration for surviving patients was 40 months (range, 5–114 months), with 1295 of 2292 patients (56.5%) surviving for over 3 years. Postoperative surveillance for recurrence was conducted following the protocols established by each participating institution.

### Serum p53 antibody (s-p53-Ab) measurement

**Fig. 2:**
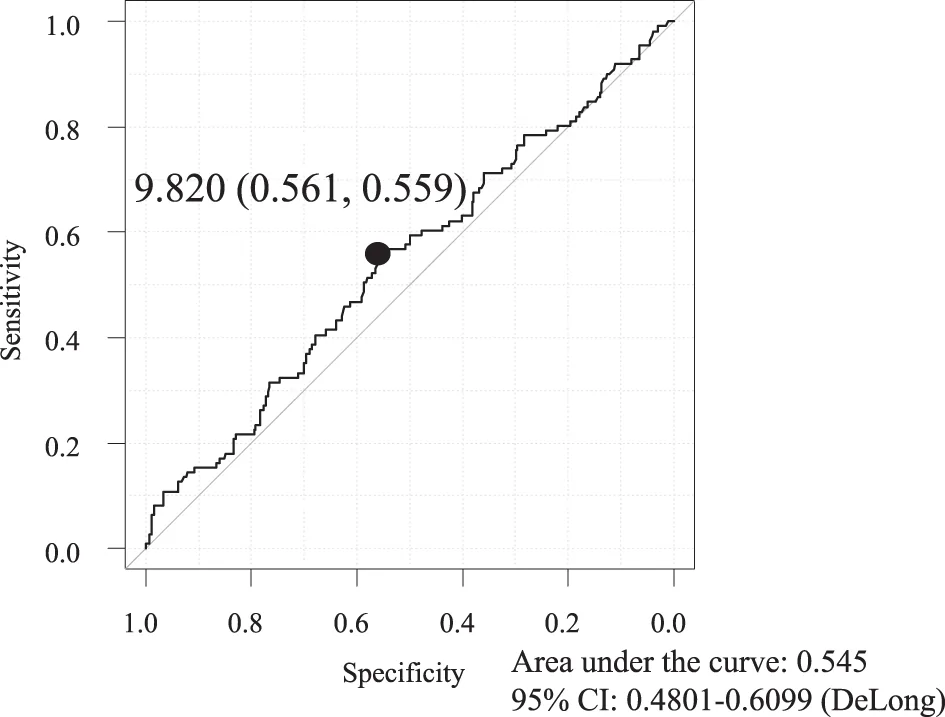
Receiver operating characteristics curve to detect the cutoff titer of s-p53-Abs to distinguish patients with a poor overall prognosis. s-p53-Abs, serum p53 antibodies

An enzyme-linked immunosorbent assay kit (MESACUP anti-p53 Test; Medical and Biological Laboratories Co., Ltd., Nagoya, Japan) was used to measure s-p53-Ab titers. The standard cutoff value of 1.30 U/mL is commonly used to distinguish patients with cancer, including those with ESCC, from healthy individuals.^11)^ Our previous study identified a titer of 9.82 U/mL as the optimal cutoff for predicting a poor prognosis, with a sensitivity of 56.1% and a specificity of 55.9% (**Fig. 2**).^7)^ This value was very close to those we previously reported in 2 different cohorts.^12,13)^ In those earlier reports, cutoff values of 10 or 13.4 U/mL were used, and values exceeding these thresholds were associated with a poor prognosis. The present study stratified s-p53-Ab-positive cases into 2 groups based on this threshold: a high-titer group (group H, s-p53-Ab ≥9.82 U/mL) and a low-titer group (group L, s-p53-Ab >1.30 U/mL to <9.82 U/mL), and their outcomes were compared. In this study, s-p53 antibody titers were measured before NAC and surgery.

### Statistical analysis

Patients were categorized into 2 treatment groups: the upfront surgery and the NAC. Fisher’s exact test was used to compare categorical variables. Overall survival (OS) was calculated from the date of surgery employing the Kaplan-Meier method, and the log-rank test was used to assess differences between survival curves. EZR software (Saitama Medical Center, Jichi Medical University, Saitama, Japan) was used for all statistical analyses.^14)^ A 2-sided p-value of <0.05 indicated statistical significance.

## Results

### Analysis of histopathological factors and treatment according to s-p53-Ab titer

Clinicopathological features and treatment strategies were compared between the high (high-titer group: s-p53-Ab ≥ 9.82 U/mL) and low s-p53-Ab titer groups (low-titer group: s-p53-Ab >1.30 to <9.82 U/mL). Variables included sex, age, tumor depth (T1/2 vs. T3/4), lymph node metastasis, distant metastasis (The location is unknown), clinical stage (cStage I–IV), and treatment modality (upfront surgery vs. NAC). The high-titer group was associated with older age, deeper tumor invasion, higher lymph node metastasis rates, more advanced clinical stage, and a greater proportion of patients receiving NAC compared with the low-titer group (**Table 1**).

**Table 1:** Comparison of clinicopathological factors between the up-front surgery group and the NAC group

Variables	Number of patients (Total = 637)	Number of L group (n = 317)	Number of H group (n = 320)	p Value^a^
Gender				
Female	100	43	57	0.157
Male	537	274	263	
Age				
<65 years old	277	150	127	0.055
≥65 years old	360	167	193	
Tumor depth				
cT1T2	326	180	146	0.006
cT3T4	311	137	174	
Nodal status				
Negative	287	158	129	0.017
Positive	350	159	191	
Distant matastasis				
Negative	611	305	306	0.842
Positive	26	12	14	
cStage				
Stage I	198	112	86	0.054
Stage II	173	87	86	
Stage III	212	91	121	
Stage IV	54	27	27	
NAC				
Absence	375	197	178	0.107
Presence	262	120	142	

^a^Fischer’s exact probability test.

*P* <0.05 statistical significance.

NAC, neoadjuvant chemotherapy

Multivariate analysis involving key prognostic factors—including age, depth of tumor invasion, lymph node metastasis, clinical stage, and treatment modality (initial surgery vs. NAC)—and s-p53-Ab titers revealed that only the depth of tumor invasion was an independent prognostic factor (**Table 2**).

**Table 2:** Multivariate analyses of risk factors for poor prognosis

Variables	Hazards ratio	95% confidence interval	Multivariate p Value^a^
Age			
<65 years old	1.046	0.785–1.393	0.761
≥65 years old			
Tumor depth			
cT1-2	1.961	1.233–3.118	0.004
cT3-4			
Nodal status			
Negative	1.155	0.686–1.946	0.588
Positive			
cStage			
cS1-2	1.417	0.757–2.655	0.276
cS3-4			
s-p53-Abs			
<9.82 U/ml, ≥1.30 U/ml	1.024	0.769–1.363	0.873
≥9.82 U/ml			
NAC			
Absence	1.067	0.792–1.437	0.670
Presence			

^a^Cox proportional hazards model.

NAC, neoadjuvant chemotherapy; s-p53-Abs, serum p53 antibodies

### S-p53-Ab positivity rates by clinical stage

**Fig. 3:**
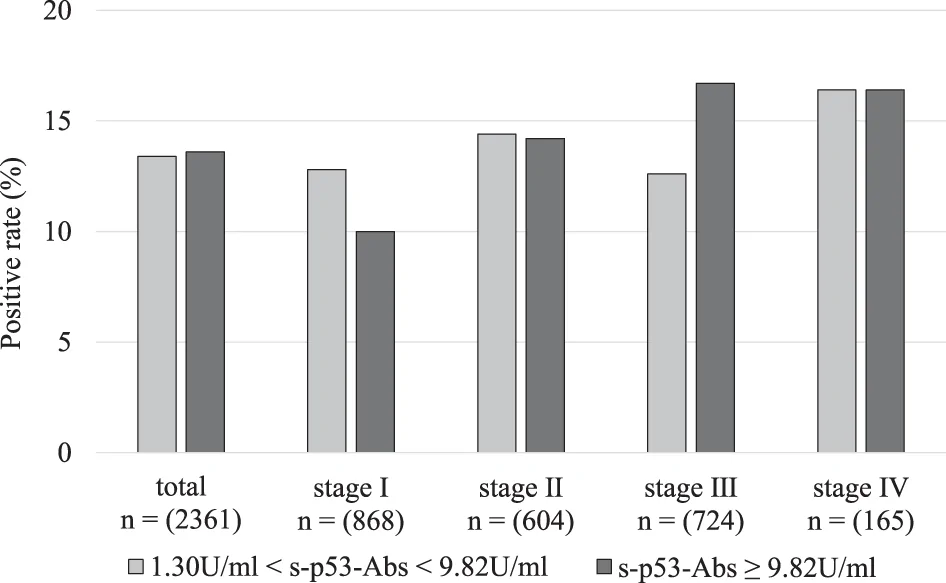
Positivity rates of s-p53-Abs according to tumor stage in 2361 patients (cutoff values of s-p53-Abs of >1.30 to <9.82 U/ml and s-p53-Abs of ≥9.82 U/ml). s-p53-Abs, serum p53 antibodies

Considering the cutoff values of 1.30 and 9.82 U/mL for s-p53-Ab, the overall positivity rates were 27.0% (637/2361) and 13.6% (321/2361), respectively. Stage-specific positivity rates for s-p53-Ab of >1.30 U/mL were cStages I (22.8% [198/868]), II (28.6% [173/604]), III (29.3% [212/724]), and IV (32.7% [54/165]). The positivity rates for the higher cutoff (s-p53-Ab ≥9.82 U/mL) were cStages I (10.0% [87/868]), II (14.2% [86/604]), III (16.7% [121/724]), and IV (16.4% [27/165]) (**Fig. 3**).

### Comparison of recurrence and survival according to s-p53-Ab titer

Among patients with positive s-p53-Ab (upfront surgery: n = 375; NAC: n = 262), recurrence and survival outcomes were compared between the high- and low-titer groups.

**Fig. 4:**
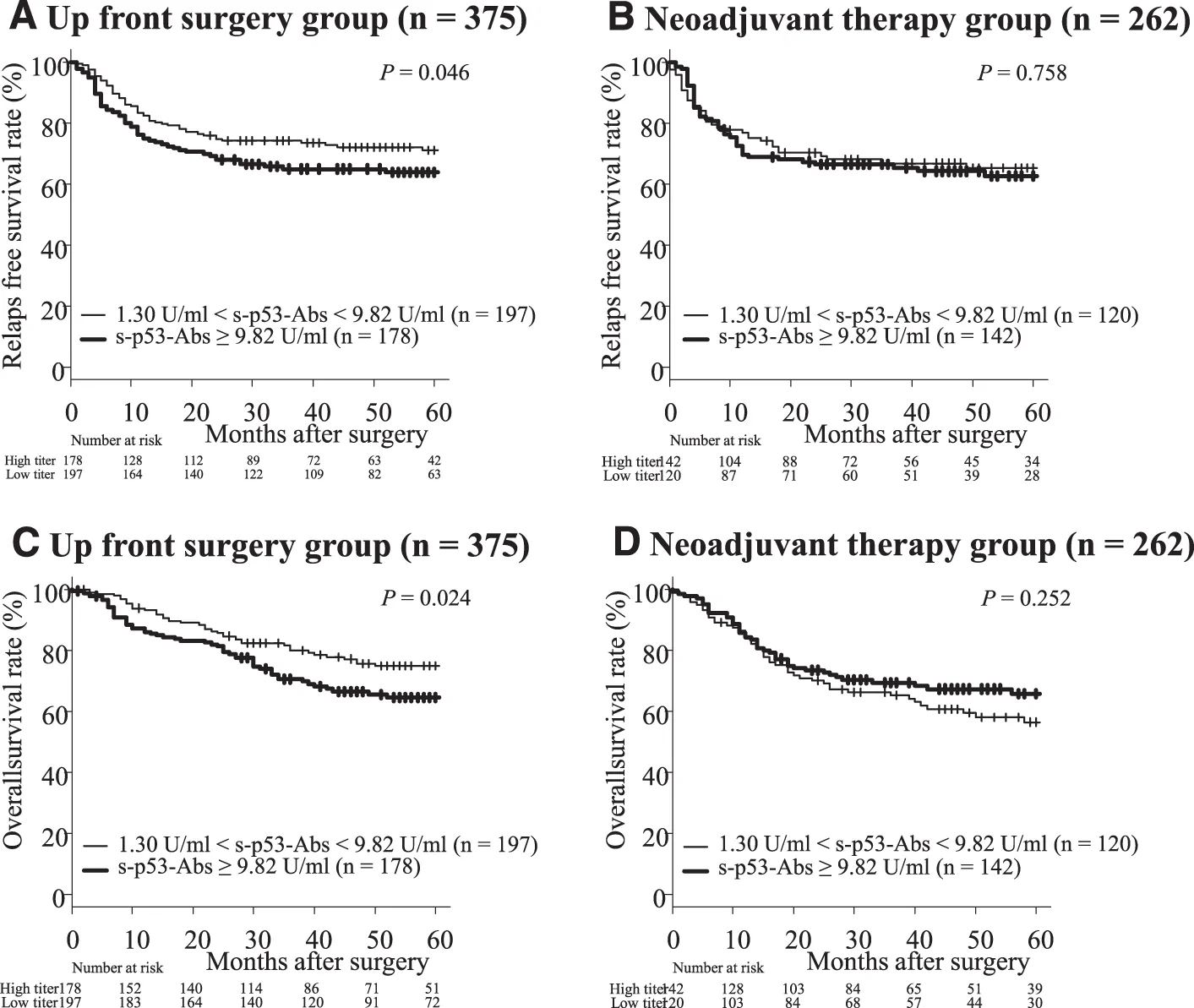
Relapse-free survival and overall survival curves according to three s-p53-Abs cutoff levels in the upfront surgery (**A** and **C**) and neoadjuvant chemotherapy (**B** and **D**) groups, respectively. The thick lines represent the survival curves of patients with high-titer s-p53-Abs levels. The thin line indicates the survival curves of patients with low-titer s-p53-Abs levels. s-p53-Abs, serum p53 antibodies

Recurrence-free survival (RFS): In the upfront surgery group, the high-titer group demonstrated a significantly higher risk of recurrence or metastasis than the low-titer group (P = 0.046; **Fig. 4A**).

No significant difference in RFS was observed between the high- and low-titer groups in the NAC group (P = 0.758; **Fig. 4B**).

OS: In upfront surgery cases, the high-titer group demonstrated significantly worse OS than the low-titer group (P = 0.024; **Fig. 4C**).

The high-titer group demonstrated better survival than the low-titer group in NAC cases, although the difference was not statistically significant (P = 0.252; **Fig. 4D**).

### Stage-specific comparison of recurrence and survival

#### Clinical stage II

**Fig. 5:**
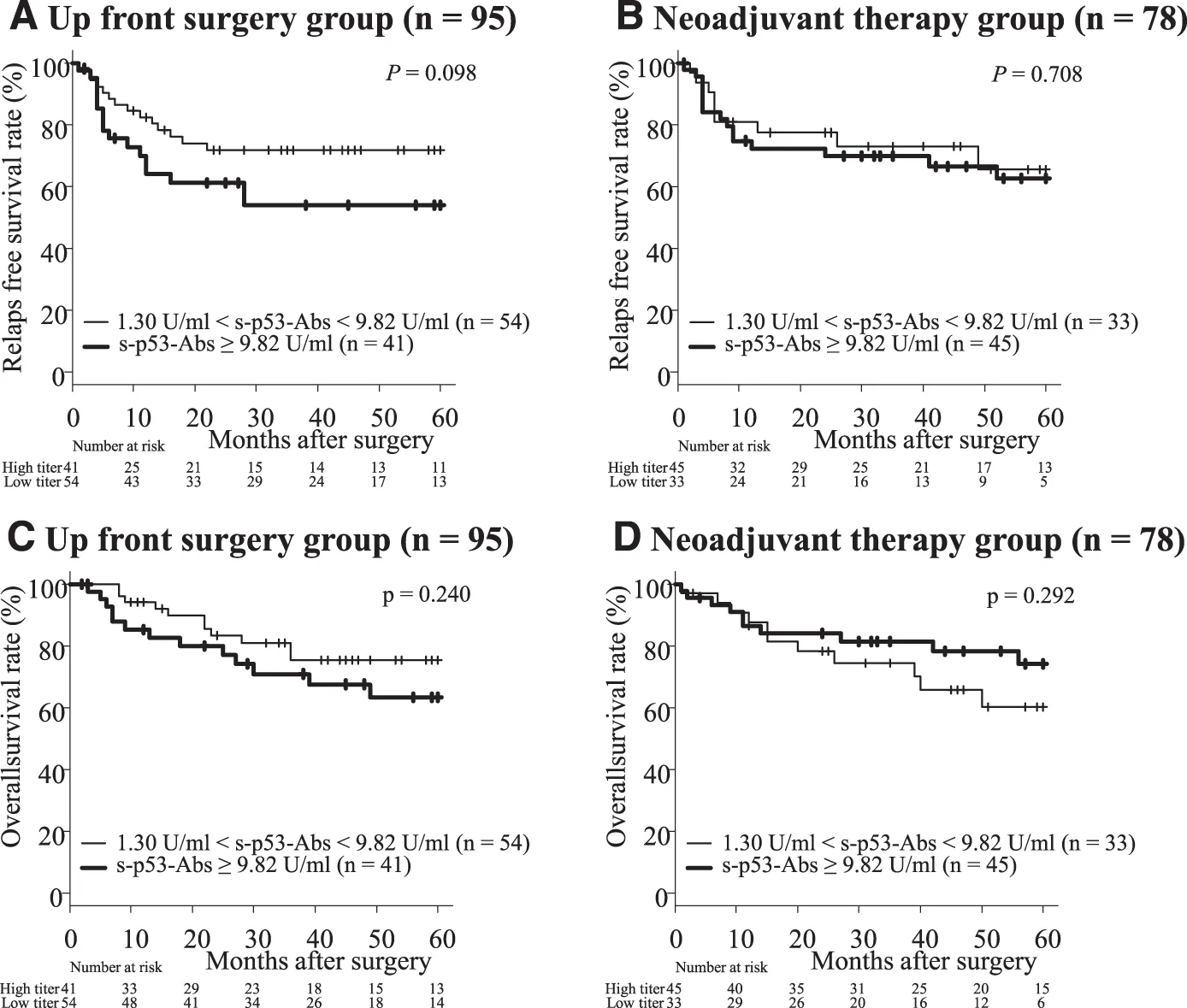
Relapse-free survival and overall survival curves according to the two s-p53-Abs cutoff levels in the upfront surgery (**A** and **C**) and neoadjuvant chemotherapy (**B** and **D**) groups, limited to cStage II. s-p53-Abs, serum p53 antibodies

Regarding RFS, in the upfront surgery group, the high-titer group demonstrated a trend toward higher recurrence and metastasis compared with the low-titer group, without statistical significance (P = 0.098; **Fig. 5A**). In the NAC group, no significant difference in RFS was found between the 2 groups (P = 0.708; **Fig. 5B**). Regarding OS, among patients undergoing upfront surgery, the high-titer group exhibited a worse prognosis than the low-titer group, although not statistically significant (P = 0.240; **Fig. 5C**). Among patients in the NAC group, the high-titer group showed a trend toward improved prognosis compared with the low-titer group, again without statistical significance (P = 0.292; **Fig. 5D**).

#### Clinical stage III

**Fig. 6:**
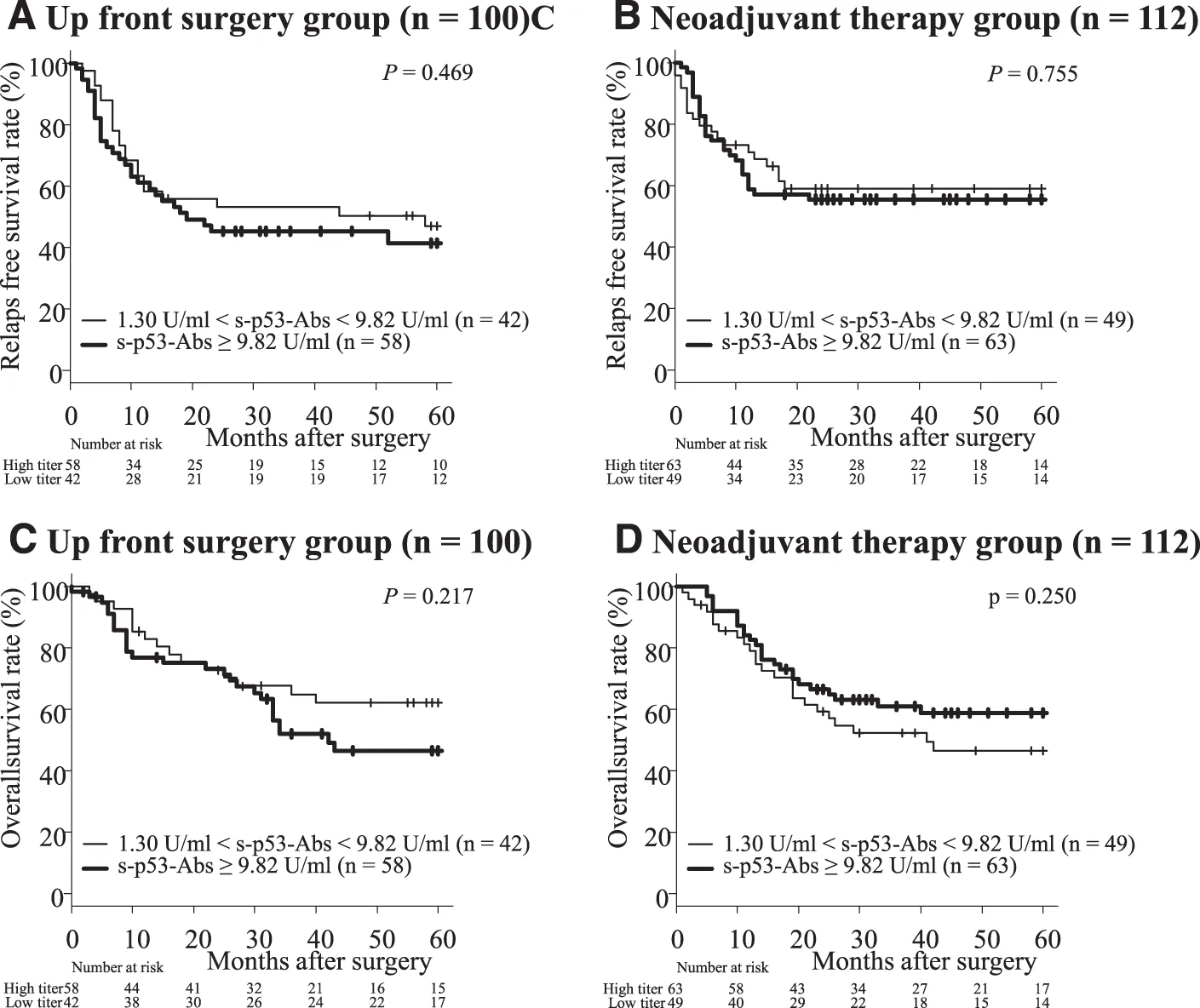
Relapse-free survival and overall survival curves according to the two s-p53-Abs cutoff levels in the upfront surgery (**A** and **C**) and neoadjuvant chemotherapy (**B** and **D**) groups, limited to cStage III. s-p53-Abs, serum p53 antibodies

Regarding RFS, in the upfront surgery group, the high-titer group demonstrated a nonsignificant tendency toward higher recurrence and metastasis rates than the low-titer group (P = 0.469; **Fig. 6A**). In the NAC group, no significant difference in RFS was observed between the 2 groups (P = 0.755; **Fig. 6B**). Concerning OS, in the upfront surgery group, the high-titer group exhibited worse OS than the low-titer group, although not statistically significant (P = 0.217; **Fig. 6C**). In patients receiving NAC, the high-titer group demonstrated a trend toward improved OS compared with the low-titer group, but the difference was not statistically significant (P = 0.250; **Fig. 6D**).

### Changing pattern of serum titers

**Fig. 7:**
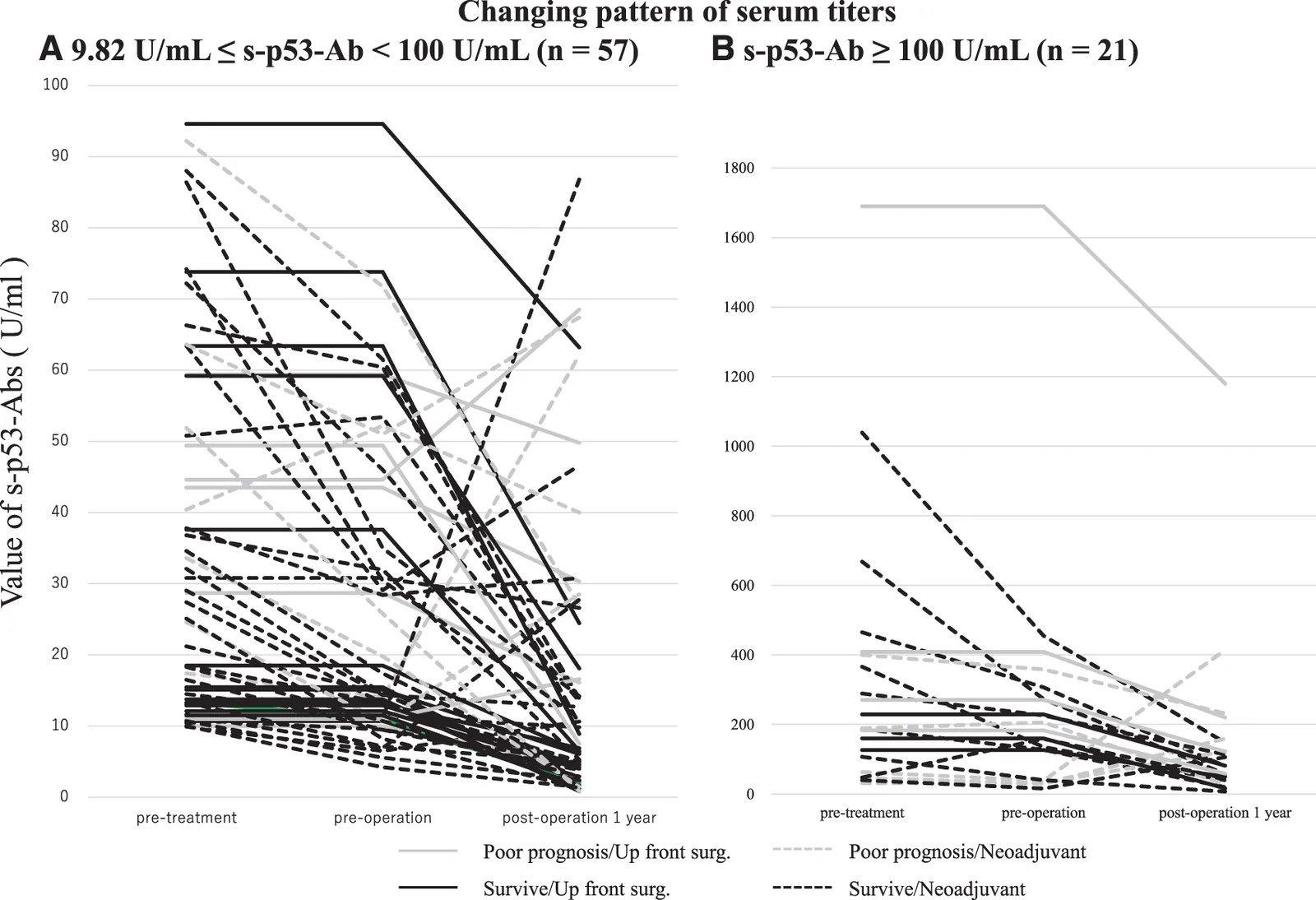
Changes in s-p53-Ab levels after NAC : Patients were stratified into 2 groups according to pretreatment s-p53-Ab levels: (**A**) 9.82 U/mL ≤s-p53-Ab <100 U/mL (n = 57) and (**B**) s-p53-Ab ≥100 U/mL (n = 21). NAC, neoadjuvant chemotherapy; s-p53-Abs, serum p53 antibodies

Changes in s-p53-Ab levels after NAC were plotted for 134 patients with available postoperative measurements at 12 months. Patients were stratified into 2 groups according to pretreatment s-p53-Ab levels: 9.82 U/mL ≤s-p53-Ab <100 U/mL (n = 57) and s-p53-Ab ≥100 U/mL (n = 21). Postoperative changes in s-p53-Ab levels are shown in **Fig. 7**. (The distinction regarding the 100 U/mL concentration here is made for the sake of clarity in notation, not for statistical reasons.)

No significant difference in poor prognosis was observed according to the presence or absence of NAC (P = 0.313). In addition, changes in s-p53-Ab levels between post-NAC and preoperative measurements (decrease vs. increase) were not significantly associated with poor prognosis (P = 0.587). In contrast, an increase in s-p53-Ab levels at 1 year after surgery was significantly associated with a higher incidence of poor prognosis (P = 0.004). However, no significant difference in 1-year postoperative changes (decrease vs. increase) was observed between patients who received NAC and those who did not (P = 0.115).

## Discussion

In this multi-institutional study, we analyzed the prognostic significance of s-p53-Ab titers in patients with s-p53-Ab-positive ESCC who were treated with NAC. The positivity rate according to clinical stage was approximately 30% and 15% in the low- and high-titer groups, respectively. No significant difference was observed between stages in either group. The consistent positivity rate, even in stage I disease, supports the reliability of the comparative analyses performed in this study.

Among patients who underwent upfront surgery, those with high titers demonstrated a significantly higher risk of recurrence compared with those with low titers. In contrast, no significant difference in recurrence risk was observed between the 2 groups in the NAC cohort, indicating a potential antitumor effect of NAC in the high-titer group. Similarly, OS analysis revealed that, in the upfront surgery group, the high-titer groups were associated with a poorer prognosis. However, the high-titer group demonstrated comparable outcomes to the low-titer group. These findings suggest that NAC may be particularly effective in patients with high s-p53-Ab titers, potentially reducing the risk of recurrence and improving long-term survival.

When outcomes were stratified according to clinical stage, particularly in stages II and III, the prognostic pattern observed in the upfront surgery group—where the high-titer group tended to have poorer outcomes—appeared to differ in the NAC cohort. Although these differences did not reach statistical significance, patients with high s-p53-Ab titers in stage II disease showed a trend toward more favorable survival compared with the low-titer group.

These findings should be interpreted with caution. One possible explanation is that the low-titer group may include tumors with low antigenicity, resulting in relatively low levels of p53 antibody production relative to tumor burden. Alternatively, it may reflect a diminished host immune response, potentially allowing more aggressive tumor proliferation.

In general, s-p53-Abs positivity has been associated with chemoresistance,^8,13,15)^ and we had previously shared this assumption. However, our current results contrast with those reports. In previous studies, the cutoff values for s-p53-Abs positivity were relatively low, which may have limited the identification of high-titer cases. Squamous cell carcinoma antigen (SCC antigen) is an important tumor marker for ESCC. Previous studies have shown that SCC antigen levels are associated with prognosis and the efficacy of chemotherapy, and that patients with elevated SCC antigen levels tend to exhibit increased resistance to treatment.^16)^ In the present study, the observation that low-titer positive cases indicated a poorer response to chemotherapy implies that previous reports may not have captured the prognostic and predictive significance of truly high s-p53-Abs titer.

Although the evaluation of perioperative changes in s-p53-Ab levels remains complex, a decrease in antibody titers was observed in most patients who underwent NAC and surgery. Changes in preoperative s-p53-Ab levels appeared to show only a limited association with poor prognosis, whereas an increase in s-p53-Ab levels at 1 year after surgery was associated with poorer outcomes.

Because changes observed at 1 year postoperatively were not significantly associated with the administration of NAC, these fluctuations may be influenced more by surgical intervention than by NAC itself. Therefore, the prognostic impact of NAC may be difficult to explain solely on the basis of perioperative changes in s-p53-Ab levels.

s-p53-Ab levels at the time of recurrence were not available for analysis and therefore could not be evaluated in the present study.

The biological mechanism underlying the favorable outcomes observed in patients with high s-p53-Ab titers who received NAC remains unclear. Previous studies have suggested that TP53 status is associated with the immune responsiveness of the tumor microenvironment, and that chemotherapy may modulate antitumor immune responses by altering the tumor microenvironment.^17)^ In addition, high s-p53-Ab titers are thought to reflect the accumulation of mutant p53 protein within tumor cells and may therefore represent a distinct biological phenotype. It is possible that such tumors exhibit greater sensitivity to chemotherapy, leading to more effective eradication of occult micrometastatic disease and improved long-term outcomes following NAC. However, the present study did not evaluate TP53 mutation status, p53 protein expression, or features of the tumor immune microenvironment. Therefore, these mechanistic interpretations remain speculative and are not directly supported by the current data. Further studies integrating serum s-p53-Ab measurements with molecular and immunological analyses of tumor tissue are warranted to clarify the biological basis underlying the favorable outcomes observed in patients with high s-p53-Ab titers.

This study had several limitations. This was a multi-institutional collaborative study; thus, detailed information regarding postoperative adjuvant chemotherapy and treatment strategies for recurrence or metastasis was unavailable, thereby limiting further analysis. In addition, data on postoperative changes in s-p53-Abs levels or their potential elevation at the time of recurrence were not collected, which precludes longitudinal assessment. Moreover, information concerning docetaxel, cisplatin and fluorouracil (DCF) therapy or immune checkpoint inhibitor-based chemotherapy, which are now included in the current Japanese treatment guidelines, was not available. Another important limitation is that molecular analyses, including TP53 mutation status, p53 immunohistochemistry, and evaluation of the tumor immune microenvironment, were not available. Therefore, the biological mechanisms underlying the association between high s-p53-Ab titers and the efficacy of NAC could not be investigated.

## Conclusion

High s-p53-Ab titers were associated with a poor prognosis in patients undergoing upfront surgery but not in those receiving NAC. Although no statistically significant differences were observed within the NAC cohort, high-titer patients tended to achieve outcomes comparable to those of low-titer patients. These findings raise the possibility that NAC may attenuate the adverse prognostic impact associated with high s-p53-Ab titers. Further prospective studies are warranted to validate this hypothesis.

## Declarations

### Data availability

The datasets generated and/or analyzed during the current study are available from the corresponding author on reasonable request.
